# Simultaneous gene silencing of *KRAS* and anti-apoptotic genes as a multitarget therapy

**DOI:** 10.18632/oncotarget.6766

**Published:** 2015-12-26

**Authors:** Kristin Werner, Franziska Lademann, May-Linn Thepkaysone, Beatrix Jahnke, Daniela E. Aust, Christoph Kahlert, Georg Weber, Jürgen Weitz, Robert Grützmann, Christian Pilarsky

**Affiliations:** ^1^ Department of Visceral, Thoracic and Vascular Surgery, TU Dresden, 01307 Dresden, Germany; ^2^ Institute of Pathology, TU Dresden, 01307 Dresden, Germany; ^3^ Department of Surgery, Universitätsklinikum Erlangen, 91054 Erlangen, Germany

**Keywords:** KRAS, apoptosis, RNAi, simultaneous gene silencing, pancreatic cancer

## Abstract

Pancreatic cancer is one of the most lethal tumor types worldwide and an effective therapy is still elusive. Targeted therapy focused against a specific alteration is by definition unable to attack broad pathway signaling modification. Tumor heterogeneity will render targeted therapies ineffective based on the regrowth of cancer cell sub-clones. Therefore multimodal therapy strategies, targeting signaling pathways simultaneously should improve treatment.

SiRNAs against *KRAS* and the apoptosis associated genes *BCLXL*, *FLIP*, *MCL1L*, *SURVIVIN* and *XIAP* were transfected into human and murine pancreatic cancer cell lines. Induction of apoptosis was measured by Caspase 3/7 activation, subG1 FACS analysis and PARP cleavage. The therapeutic approach was tested in a subcutaneous allograft model with a murine cancer cell line.

By using siRNAs as a systematic approach to remodel signal transduction in pancreatic cancer the results showed increasing inhibition of proliferation and apoptosis induction *in vitro* and *in vivo*. Thus, siRNAs are suitable to model multimodal therapy against signaling pathways in pancreatic cancer. Improvements in *in vivo* delivery of siRNAs against a multitude of targets might therefore be a potential therapeutic approach.

## INTRODUCTION

Pancreatic ductal adenocarcinoma (PDAC) is one of the most lethal tumors worldwide, because of its late diagnosis and poor therapy response [1]. Despite recent advances in the therapy of PDAC, a cure is an unachieved goal yet. an unachieved goal [2, 3].

Activating *KRAS* mutations are already found in precursor lesions and nearly universal (> 95%) in tumors. It is considered to be the central oncogene of PDAC, because it promotes proliferation, migration, apoptosis evasion and it inhibits differentiation [4–7]. Targeting *KRAS* seems to be central for the treatment of PDAC. However, there are no effective small molecule inhibitors clinically available [8, 9].

PDAC cells are characterized by inhibition of apoptosis signaling, which seems to be responsible for the insufficiency of most current therapies [6, 7, 10]. Extrinsic death signals are blocked by overexpressed decoy receptors and redirection to non-apoptotic pathways. DISC formation is weak and activation of the initiator caspase-8 is repressed by Flip [11]. Activation of the mitochondrial pathway is necessary for an effective induction of the caspase cascade [12]. Here, the balance between pro- and anti-apoptotic proteins is crucial. In PDAC the Bcl2 family members Bclx and Mcl1 show high expression levels of their anti-apoptotic splice variants promoting the resistance towards death signals and chemotherapeutics [12–15]. Caspases are blocked by inhibitors of apoptosis proteins (IAPs) like Xiap and Survivin downstream of this extrinsic and intrinsic apoptosis induction [16, 17]. Furthermore, overexpression of these five anti-apoptotic genes in PDAC is facilitated by mutated *KRAS* via feedback loops or by inhibition of their antagonists [18–20].

SiRNA treatment to repress gene expression has become a standard technology to interrogate gene function in a highly specific manner [21, 22]. However, few data on simultaneous gene silencing (SGS) are available [23–26]. SGS enables the knockdown of more than one gene, leading to new approaches for pathway targeting and can simulate precision medicine [26]. We have analyzed the possibilities of pathway targeting using SGS against six different genes, encoding five proteins of the apoptosis pathway (Bclx_L_, Flip, Mcl1_L_, Xiap, Survivin) and KRas in a panel of human and murine pancreatic cancer cell lines. We were able to confirm that the combination of siRNAs initiates a specific knockdown of the selected target genes, which is highly effective in triggering apoptosis and inhibiting proliferation *in vitro* and *in vivo*.

## RESULTS

### Heterogeneous expression of SGS proteins in human and murine pancreatic cancer cell lines

**Figure 1:**
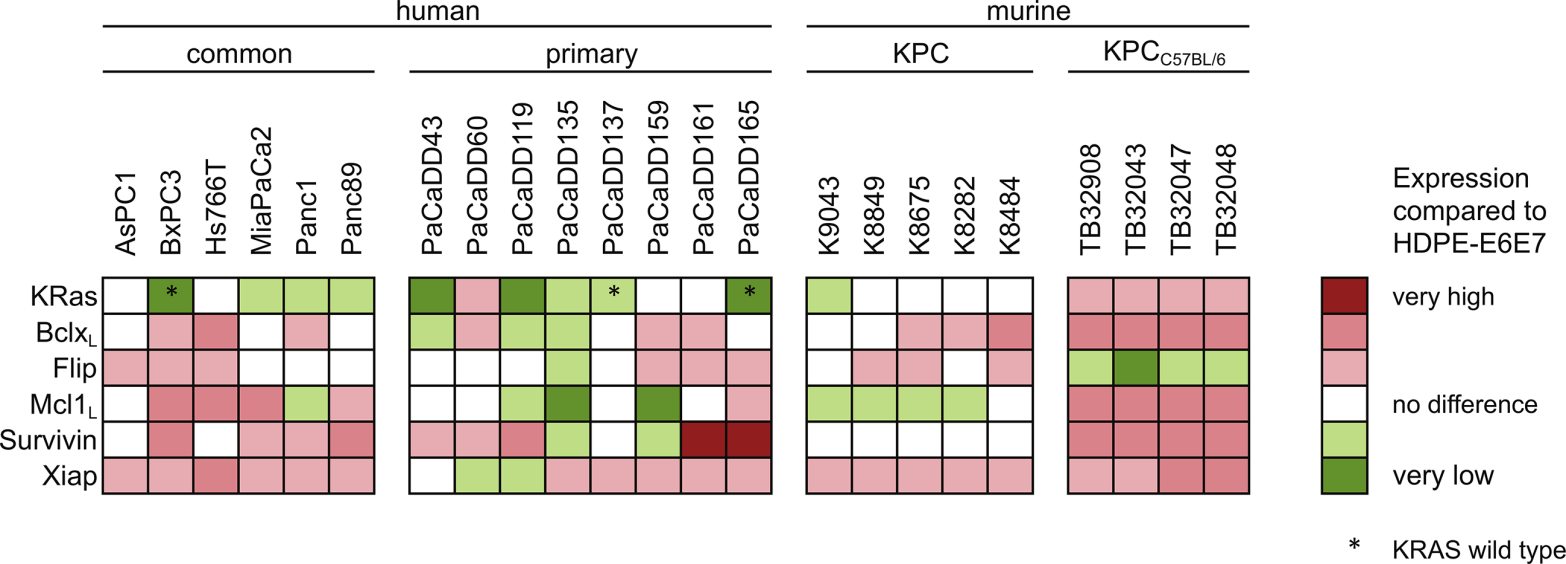
Expression of KRas and apoptosis associated proteins in PDAC cell lines Cell lysates of standard and primary human pancreatic cancer cell lines and from the *KPC* mouse model were subjected to Western blot analysis. Expression levels of Bclx_L_, Flip, KRas, Mcl1_L_, Survivin and Xiap were analyzed compared to the human non-tumorous, epithelial pancreatic duct HDPE-E6E7 cell line.

The expression of KRas, Bclx_L_, Mcl1_L_, Flip, Survivin and Xiap was evaluated by Western blot analysis and normalized to the non-tumorous cell line HDPE- E6E7 from pancreatic duct epithelium (Figure 1, Figure S8). The anti-apoptotic proteins Flip, Survivin and Xiap were overexpressed in pancreatic cancer. The long splice variants of Bclx and Mcl1 with anti-apoptotic potential have also shown a higher expression regarding to the non-tumorous control cell line. The expression level of KRas in HDPE-E6E7 cells was comparable to the other cell lines, however activating mutations of *KRAS* can be found in most of the human cell lines, in the standard cell lines as well as in the primary cell lines established from PDAC tissue (detailed overview see Table S1). Altogether the expression pattern seems to be notably heterogeneous and is reflected in the *KPC* mouse model cell lines.

### SiRNAs against human and murine target genes

To establish siRNAs targeting the anti-apoptotic genes *BCLXL, FLIP, MCL1L, SURVIVIN, XIAP*, and *KRAS*, two cell lines from human and murine origin were chosen. MiaPaCa2 and Panc1 cells were treated for 72 h with high concentrations (72 nM) of the siRNAs directed against the human genes. Their orthologous genes were silenced in the murine cell lines K9043 and TB32047. Compared to the negative control (NC) a reduction of transcript levels by 40–99% was determined in all cell lines measured by qRT-PCR. The knockdowns on protein level were validated by Western blots for every experiment (Figure S1).

To examine the proliferation inhibition and apoptosis induction caused by silencing of these target genes, the cells were counted and subG1 fractions were analysed by flow cytometry (Figure S2). Reductions in cell counts (by 22–87%) and increased subG1 fractions (12–51%), reflecting the apoptosis induction, were observed after single knockdown with high concentrated siRNAs.

### Simultaneous gene silencing (SGS6) of *BCLXL*, *FLIP*, *KRAS*, *MCL1L*, *XIAP* and *SURVIVIN* as multitarget therapy

**Figure 2:**
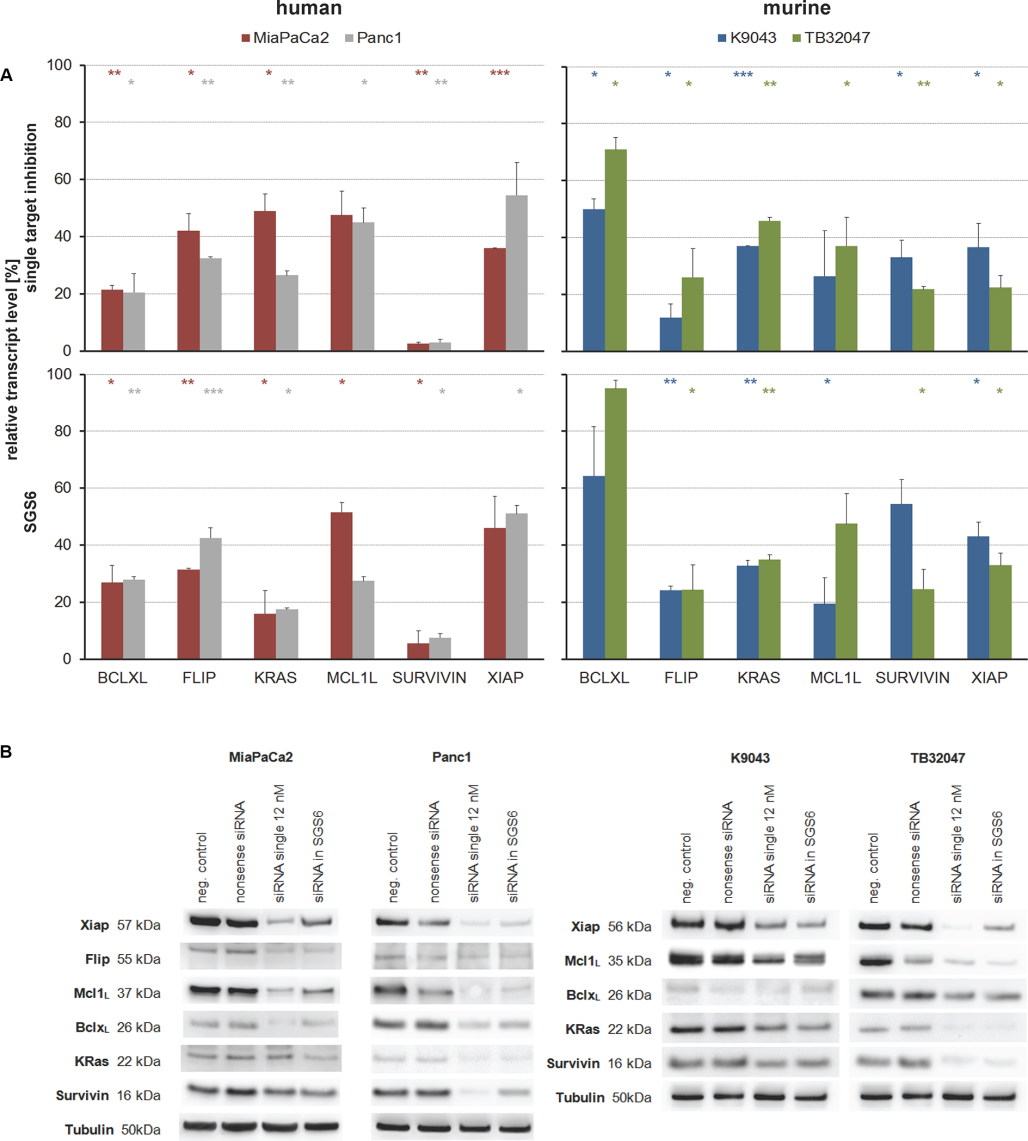
Target inhibition by single and combined, low-concentration siRNA treatment Human and murine PDAC cell lines were transfected with 12 nM siRNAs per target gene for 72 h. For single target inhibition the siRNA was filled up with nonsense siRNA to 72 nM. For simultaneous gene silencing (SGS6) six siRNAs, each with a concentration of 12 nM, were used. (**A**) On transcriptional level knockdowns were confirmed by qRT-PCR relative to their negative control (*n* ≥ 2; *p ≤ 0,05, **p ≤ 0,01, ***p ≤ 0,001). (**B**) On protein level silencing was always confirmed by Western blots relative to Tubulin as housekeeping protein.

**Figure 3:**
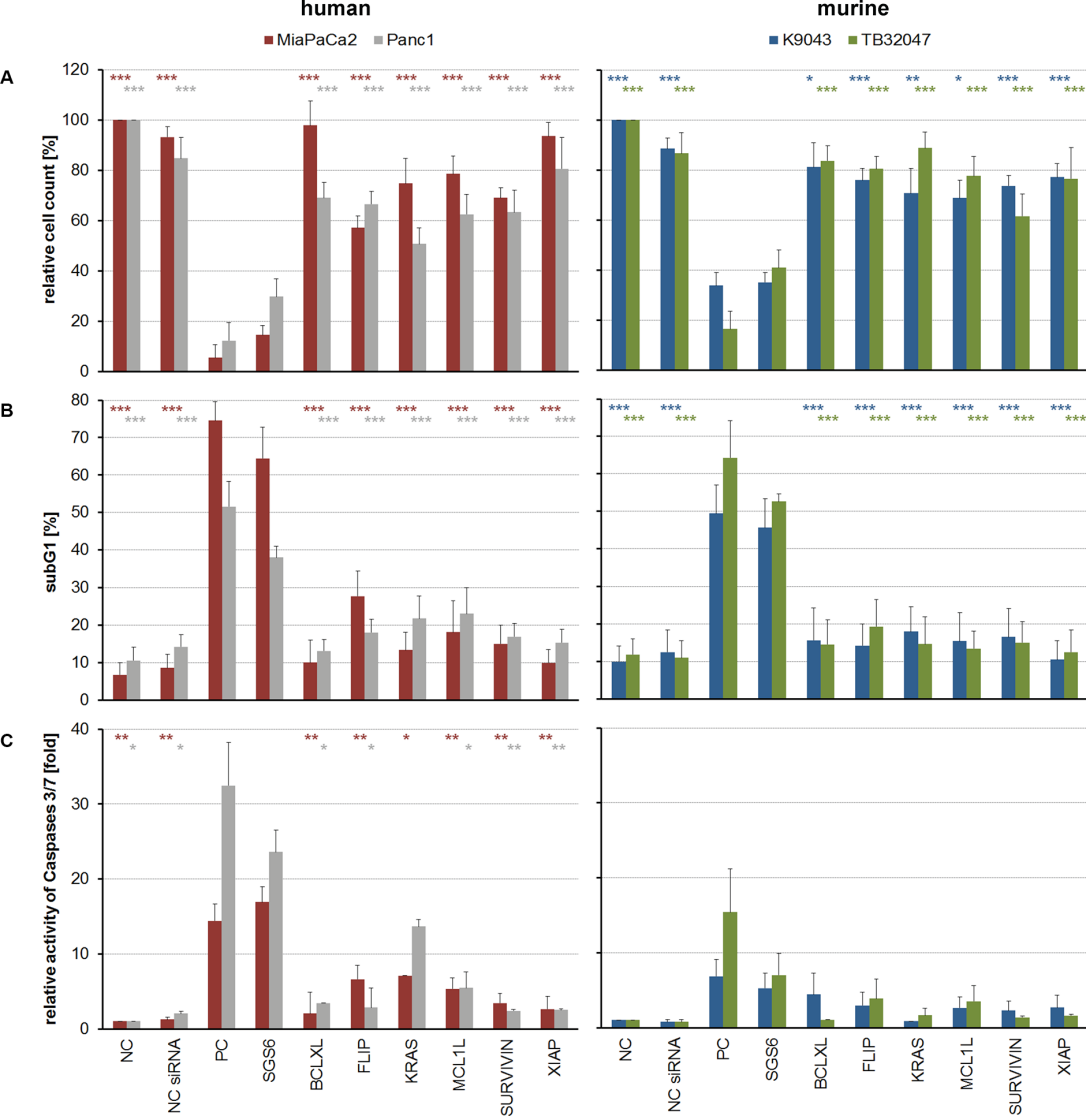
Cellular effects caused by single and combined target inhibition with low-concentrated siRNA (**A**) Adherent cells were counted in relation to their negative control (NC). (**B**) For evaluation of apoptosis induction subG1 fractions were determined by cell cycle analysis and (**C**) Caspase assays were performed. An unpaired Student's *t*-test was used to calculate the differences of the NCs or single-targeted cells in relation to the SGS6 samples (*n* ≥ 3; *p ≤ 0,05, **p ≤ 0,01, ***p ≤ 0,001).

**Figure 4:**
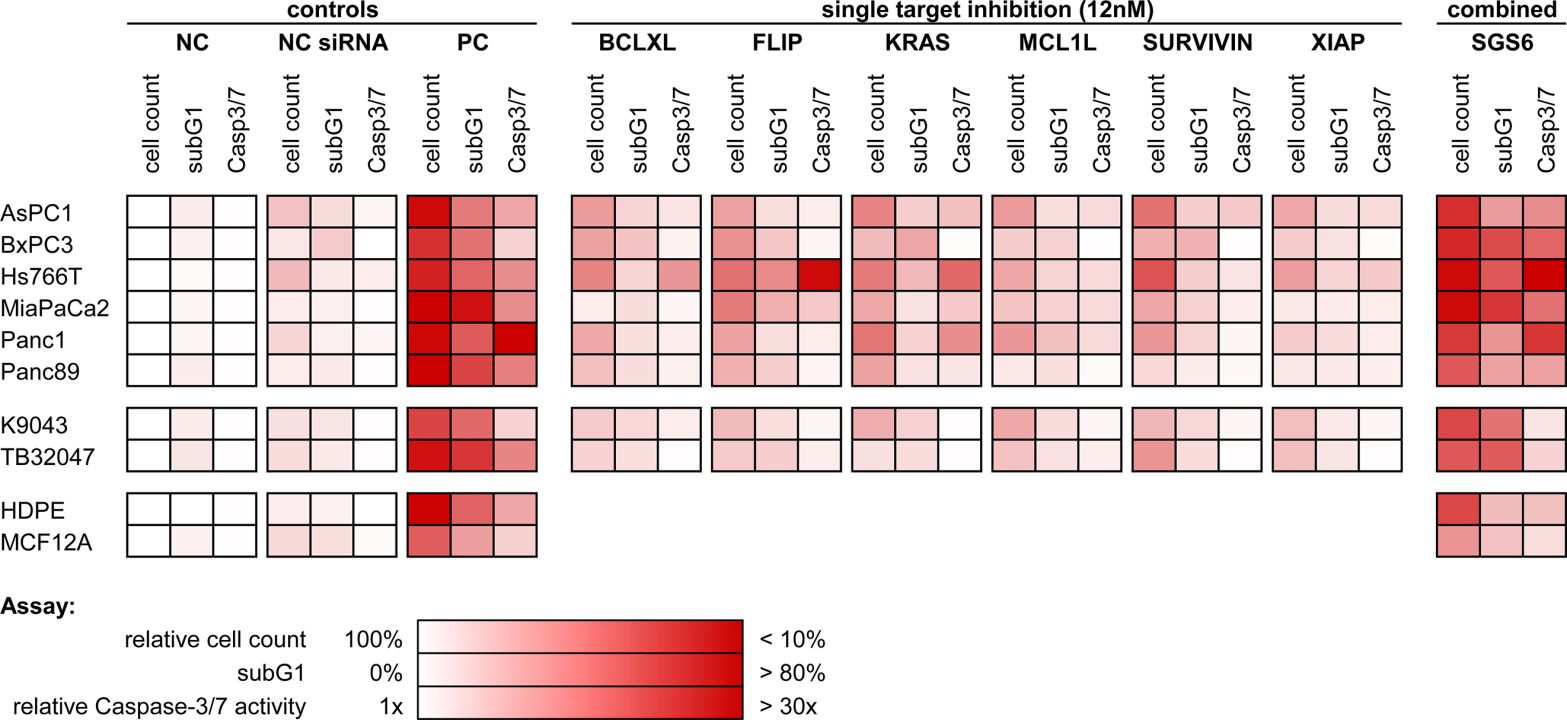
Overview of the SGS6 therapeutic effectiveness Additional PDAC cell lines were transfected analogously with SGS6 and single siRNAs. Cellular effects like general proliferation inhibition and apoptosis induction were confirmed by three assays: cell count relative to their negative control (NC), subG1 FACS analysis and relative activity of Caspases 3 and 7 (*n* ≥ 3).

As a therapeutic approach, independent of the heterogeneous protein expression (Figure 1), all six genes were silenced simultaneously with low dose concentrations of each siRNA. Knockdowns were confirmed in low dose single target inhibition experiments (12 nM target-specific siRNA + 60 nM nonsense siRNA) and in the SGS6 treatment (12 nM per all six target genes). Additionally, low dose siRNAs also decreased mRNA and protein expression levels 72 h after transfection (Figure 2). While single target inhibition with these concentrations showed only slight effects, the combined application caused a strong decrease in relative cell number of about 59–85%. Apoptosis induction determined by subG1 analysis demonstrated that 38–64% of the cells showed DNA fragmentation. Moreover, an increased activity of the Caspases 3 and 7 was observed in the range of 5–32-fold compared to the negative control (Figure 3). SGS6 was the most efficient combination to induce proliferation inhibition/apoptosis compared to other mixtures of siRNAs (Figures S3, S5 and S6). Additional human cell lines were tested for SGS6 effectiveness (Figure 4). These cells showed an inhibition of proliferation and apoptosis induction compared to the positive control. The heterogeneity of expression levels of a single target gene (Figure 1) did not influence the effect of SGS6. To investigate if SGS6 might have an effect on normal cells the non-tumorous pancreatic duct cell line HDPE-E6E7 and the non-tumorous breast cell line MCF12A were transfected likewise. Interestingly, whereas the cell number was reduced, induction of apoptosis was comparatively low.

Different combinations of these target genes were investigated (Figures S3, S5 and S6). A mix of the siRNAs targeting the apoptosis associated genes showed PARP cleavage, which was increased by the addition of *KRAS* in our SGS6.

For validation, other human siRNAs, specific for the six target genes, were established and the effectiveness of the SGS6 therapy was retested in MiaPaCa2 and Panc1 cells. In both cell lines induction of apoptosis and reduction of proliferation could be confirmed (Figure S4).

### Verification of the SGS6 therapy in subcutaneous mouse models *in vivo*

**Figure 5:**
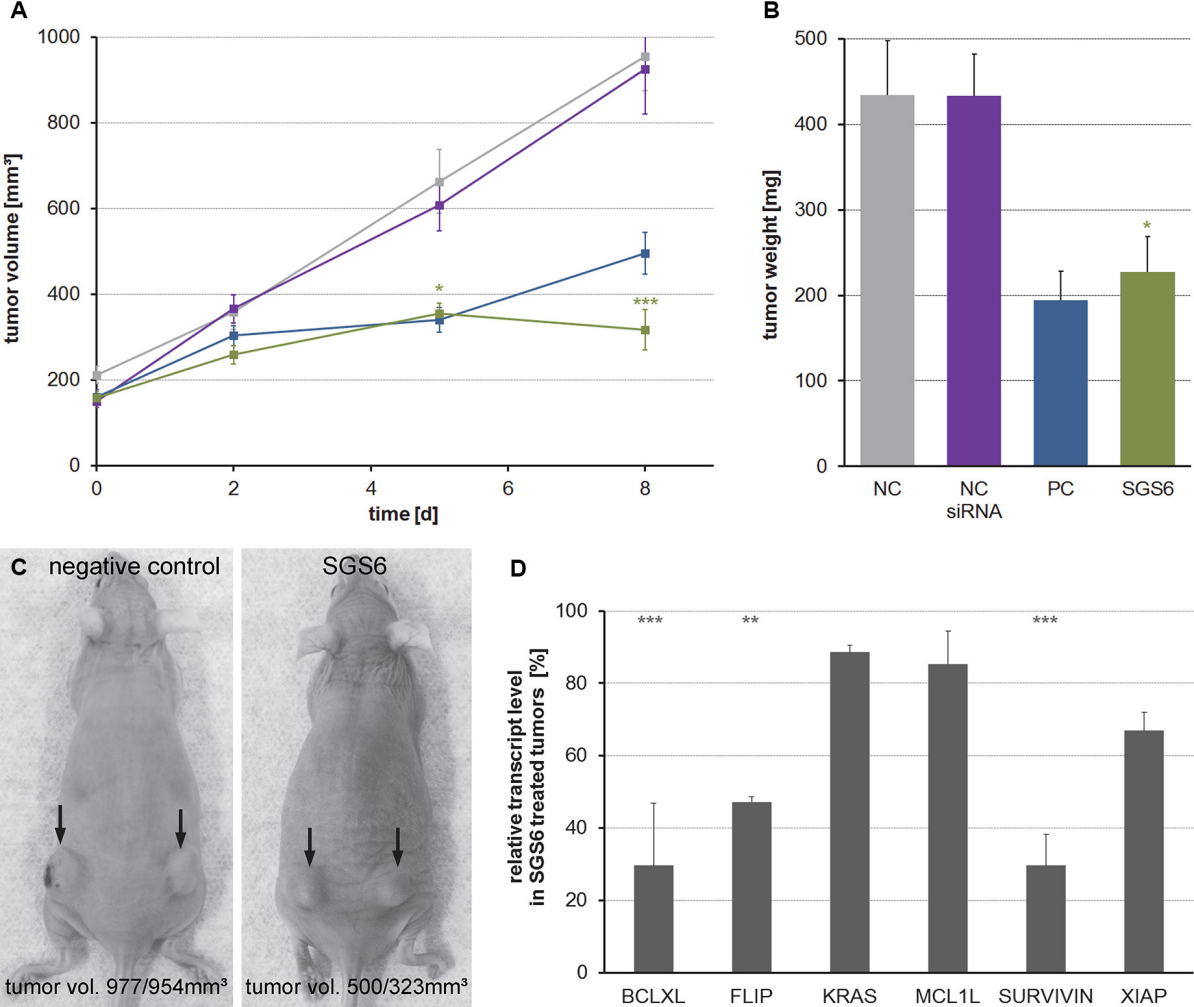
Treatment of subcutaneous TB32047-allografts with the SGS6 therapy Subcutaneous, bilateral TB32047 tumors of NMRI^nu/nu^ mice were treated daily with 10 μg of *in vivo* jet-PEI complexed SGS6 siRNA for 8 d (11–14 tumors per group). (**A**) Tumor volumes were measured during therapy and (**B**) their weight was analyzed after preparation at day 9. Means ± standard errors are shown and an unpaired Student's *t*-test was used to compare the differences between the negative control (NC) and the SGS6 treated tumors. (**C**) Exemplary photos are depicted. (**D**) Gene silencing in SGS6 treated tumors was confirmed by qRT-PCR related to the tumors treated with nonsense siRNA (*p ≤ 0,05, **p ≤ 0,01, ***p ≤ 0,001).

To verify the *in vitro* results SGS6 therapy was applied to murine and human subcutaneous tumors. TB32047, derived from *KPC* tumors, or MiaPaCa-2 cells were implanted subcutaneously in both flanks of immune-deficient NMRI^nu/nu^ mice, which consequently produced fast growing tumors. After treatment with intratumoral application of SGS6 siRNA complexed with jetPEI (11–14 tumors per group) the growth rate of the SGS6-treated tumors was significantly decreased compared to tumors treated with negative control siRNA. As a consequence the weight of the excised tumors was also lower (Figure 5A–5C, Figure S9). SiRNA-induced gene silencing was confirmed by qRT-PCR (Figure 5D). Within the tumors treated with SGS we observed more ductal structures, indicating a differentiation of the tumor cells *in vivo* (Figure S7).

## DISCUSSION

Conventional cancer chemotherapy is limited due to its toxicity in normal tissue. In cancers other than PDAC precision medicine has improved patients life and survival, because of the targeting of genetic alterations, which render the tumor susceptible to a specific treatment [27, 28]. However, based on the results of the TCGA and the ICGC efforts, a vast number of patients with PDAC might not be suitable for precision medicine as appropriate drugs are missing. Moreover, mutations in key cancer pathways might not occur in a single gene, but in different genes with variable frequencies [4]. The knowledge of tumor heterogeneity and the concept of hallmarks of cancer indicates that cancer treatment should not be reduced to a therapy of key regulators, but that cancer is a pathway disease and should be managed accordingly [29]. Therefore, we hypothesized that the combination of a key oncogene (*KRAS*) and additional pathway perturbation might be highly effective in killing cancer cells.

The apoptotic signaling pathway was chosen because of its well-known dependence on the equilibrium of pro- and anti-apoptotic factors [30]. We could demonstrate that single treatment of cells can be effective, if the cells are sensitive (*FLIP* knockdown in MiaPaCa2 and Hs766T). However, treatment of cells using the SGS6 pathway approach is highly effective in a broad range of pancreatic cancer cells *in vitro* and *in vivo*, despite their heterogeneous protein expression. Also, targeting six genes is more effective in triggering of apoptosis and inhibition of proliferation compared to the knockdown of a single or other combinations of genes. Therefore, simultaneous gene silencing offers the ability to treat signaling pathways in cancer with or without additional targeting of key regulators.

The basic toxicity of siRNAs are well studied and advances in *in vivo* delivery of siRNAs might enable treatment of patients without the development of small molecules for each target structure, therefore shortening the development cycle of cancer therapeutics [31–34]. Moreover, reducing the concentration of each siRNA in the mix might also reduce sequence specific side effects without reduction of efficacy.

In conclusion, apoptotic resistance and the heterogeneity of pancreatic tumors have been outsmarted by a widespread normalization of overexpressed anti-apoptotic genes and the inhibition of *KRAS*. By silencing of the activated *KRAS* and members of the apoptotic resistance pathway, we were able to induce apoptosis and proliferation inhibition in a broad spectrum of pancreatic cancer cells with our SGS6, *in vitro* as well as *in vivo*.

## MATERIALS AND METHODS

### Cell culture

The human pancreatic cancer cell lines AsPC1, BxPC3, MiaPaCa2 and Panc1 were obtained from American Type Culture Collection. The Hs766T cell line was kindly provided by Tatjana Crnogorac-Jurcevic (Centre for Molecular Oncology, Barts Cancer Institute, UK) and Panc89 by Holger Kalthoff (University of Schleswig-Holstein, Kiel, Germany). The HDPE-E6E7 were kindly provided by Ming Tsao, (University of Toronto, Canada) and represent immortal, but non-tumorous pancreatic duct epithelial cells. The primary cell lines (PaCaDD) were established with the Dresden Outgrowth protocol [35]. Cell lines from pancreatic cancer of the genetic engineered *KPC* mouse model were provided by David Tuveson (Cold Spring Harbor Laboratory, USA). All cell lines were cultured in a humidified atmosphere containing 5% CO_2_ at 37°C. The referenced media are listed in the Supplementary Materials (Table S2).

### Transfection of siRNAs and simultaneous gene silencing (SGS) assay

All cell lines were seeded at a density between 3–5 × 10^4^ cells/well and were transfected with siRNAs using Oligofectamine (Invitrogen, Karlsruhe, Germany) according to the manufacturer's protocol. In each transfection 72 nM of siRNAs were used in total. 12 nM of siRNA were applied per target gene, filled up with nonsense siRNA, in low-dose and combined transfections for simultaneous gene silencing (SGS). The target sequences shown in Table S3 were used with 3′-dTdT overhangs. Regarding *BCLX* and *MCL1*, the siRNAs target only the transcripts which code for the long forms of the proteins with anti-apoptotic functions [13]. As negative control we used Allstars siRNA (Qiagen, Hilden, Germany), as positive control siRNA targeting *KIF11*/Eg5, an essential cytoskeletal component. After incubation for 72 h the cells were harvested for flow cytometry and a caspase activity assay to determine the apoptosis rate. Gene silencing was confirmed by qRT-PCR and Western blot.

### Quantitative reverse transcription-PCR (qRT-PCR)

Total RNA was isolated with the NucleoSpin II-Kit (Macherey-Nagel, Düren, Germany) according to the manufacturer's instructions. Reverse transcription into cDNA was realized using the High Capacity cDNA Reverse Transcription Kit (Applied Biosystems, Foster City, USA). Transcript amounts of the targets and the reference genes *ACTB* and *GAPDH* were determined by qRT-PCR using the Power SybrGreen PCR Master Mix (LifeTechnologies, Carlsbad, USA) according to the manufacturer's protocol. All primers are listed in Table S4.

### Western blotting

Cells were washed and lysed in RIPA buffer. After incubation for 10 min at 90°C the denatured samples were separated using the NuPAGE SDS-PAGE Gel System (Invitrogen, Karlsruhe, Germany) and then transferred to a nitrocellulose membrane (Hybond ECL, GE Healthcare, Munich, Germany). After blotting with 5% skimmed milk powder in TBSTween 0,1% the membranes were probed with primary antibodies shown in Table S5. Subsequently the following secondary HRP-linked antibodies were used 1:1000: Anti-mouse IgG (#7076) and Anti-rabbit IgG (#7074, CellSignaling, Danvers, USA). By Immobilon Western Chemiluminescent HRP Substrate (Millipore, Billerica, USA) the chemiluminescence could be visualized and detected with the G:Box Chemi XT4 (Syngene, Bangalore, India).

### Determination of the relative cell count

The reduction of the cell number reflects proliferation inhibition and apoptosis. The treated, adherent cells were harvested by trypsinization according to standard cell culture procedures and counted by the TC-20 Automated Cell Counter (BIORAD, Hercules, USA). The cell numbers were normalized to the media controls (NC).

### Determination of apoptosis by flow cytometry and CaspaseGlo 3/7

For cell cycle analysis the cells were harvested 72 h after transfection and fixed with 70% ethanol overnight at 4°C. Prior to analysis the samples were washed with PBS, resuspended and treated with 0,1 mg/ml RNase A for 1 h at 37°C. Following to an incubation with 50 μg/ml propidium iodide the samples were analyzed with the flow cytometer FACS Calibur (Beckton Dickinson, Heidelberg, Germany). After doublet discrimination at least 1 × 10^4^ single cells were assayed regarding their subG1 fraction, which represents dead cells.

Confirming apoptosis induction the activity of Caspases 3 and 7 was measured using the Caspase-Glo 3/7 Assay (Promega, Mannheim, Germany) according to the manufacturer's protocol. 72 h after transfection cell pellets with a maximum of 2 × 10^4^ cells in the media controls were frozen and the results were calculated on the basis of the cell numbers. Normalization followed to the media controls.

### *In vivo* experiments

Animal studies were conducted in accordance with the German Animal Protection Law. Ten-week-old female NMRI^nu/nu^ mice, obtained from the animal facility of the University of Dresden, were held under standardized pathogen-free conditions with *ad libitum* access to food and water. 1 × 10_6_ TB32047 or MiaPaCa2 cells, resuspended in 50 μl PBS, were injected bilateral into the flanks. Using a digital caliper the established tumors were measured and the tumor volumes were calculated with the following formula: V = 1/6 × π × length × width × 1/2 (length + width). After 12 – 28 d the mice were subdivided into four groups with the same tumor volume (TB32047: 150 – 200 mm^³^, MiaPaCa2: 250 – 300 mm^³^). The tumors were treated by intratumoral injections of 10 μg of *in vivo*-jetPEI (Polyplus, Strasbourg, France) complexed siRNAs. Additional to the group of SGS6 therapy, one group remained untreated, one received nonsense Allstars siRNA (Qiagen, Hilden, Germany) and one was treated with Eg5 siRNA as positive control for the local transfection effectiveness. Mice were sacrificed one day after the last treatment and the tumors were prepared for weight measurement and mRNA analysis. The experiments were performed twice with 11 to 14 tumors per group in total.

### Statistical analysis

Data are presented as mean ± standard deviation. All were from at least three independent experiments and subjected to paired *t*-tests.

## SUPPLEMENTARY MATERIALS FIGURES AND TABLES



## References

[1] Siegel R, Ma J, Zou Z, Jemal A (2014). Cancer statistics, 2014. CA Cancer J Clin.

[2] Von Hoff DD, Ervin T, Arena FP, Chiorean EG, Infante J, Moore M, Seay T, Tjulandin SA, Ma WW, Saleh MN, Harris M, Reni M, Dowden S (2013). Increased survival in pancreatic cancer with nab-paclitaxel plus gemcitabine. N Engl J Med.

[3] Conroy T, Desseigne F, Ychou M, Bouche O, Guimbaud R, Becouarn Y, Adenis A, Raoul JL, Gourgou-Bourgade S, de la Fouchardiere C, Bennouna J, Bachet JB, Khemissa-Akouz F (2011). FOLFIRINOX versus gemcitabine for metastatic pancreatic cancer. N Engl J Med.

[4] Waddell N, Pajic M, Patch AM, Chang DK, Kassahn KS, Bailey P, Johns AL, Miller D, Nones K, Quek K, Quinn MC, Robertson AJ, Fadlullah MZ (2015). Whole genomes redefine the mutational landscape of pancreatic cancer. Nature.

[5] Ryan DP, Hong TS, Bardeesy N (2014). Pancreatic adenocarcinoma. N Engl J Med.

[6] Jones S, Zhang X, Parsons DW, Lin JC-H, Leary RJ, Angenendt P, Mankoo P, Carter H, Kamiyama H, Jimeno A, Hong S-M, Fu B, Lin M-T (2008). Core signaling pathways in human pancreatic cancers revealed by global genomic analyses. Science.

[7] Werner K, Rückert F, Wehrum D, Samm N, Saeger H-D, Grützmann R, Pilarsky C (2011). Role of apoptosis dysregulation in pancreatic cancer. Cancer Reports.

[8] Maurer T, Garrenton LS, Oh A, Pitts K, Anderson DJ, Skelton NJ, Fauber BP, Pan B, Malek S, Stokoe D, Ludlam MJ, Bowman KK, Wu J (2012). Small-molecule ligands bind to a distinct pocket in Ras and inhibit SOS-mediated nucleotide exchange activity. Proc Natl Acad Sci USA.

[9] Appels NM, Beijnen JH, Schellens JH (2005). Development of farnesyl transferase inhibitors: a review. Oncologist.

[10] Collins MA, Pasca di Magliano M (2013). Kras as a key oncogene and therapeutic target in pancreatic cancer. Frontiers in physiology.

[11] Haag C, Stadel D, Zhou S, Bachem MG, Moller P, Debatin KM, Fulda S (2011). Identification of c-FLIP(L) and c-FLIP(S) as critical regulators of death receptor-induced apoptosis in pancreatic cancer cells. Gut.

[12] Hinz S, Trauzold A, Boenicke L, Sandberg C, Beckmann S, Bayer E, Walczak H, Kalthoff H, Ungefroren H (2000). Bcl-XL protects pancreatic adenocarcinoma cells against CD95- and TRAIL-receptor-mediated apoptosis. Oncogene.

[13] Moore MJ, Wang Q, Kennedy CJ, Silver PA (2010). An alternative splicing network links cell-cycle control to apoptosis. Cell.

[14] Wei SH, Dong K, Lin F, Wang X, Li B, Shen JJ, Zhang Q, Wang R, Zhang HZ (2008). Inducing apoptosis and enhancing chemosensitivity to Gemcitabine via RNA interference targeting Mcl-1 gene in pancreatic carcinoma cell. Cancer Chemoth Pharm.

[15] Akgul C (2009). Mcl-1 is a potential therapeutic target in multiple types of cancer. Cell Mol Life Sci.

[16] Lopes RB, Gangeswaran R, McNeish IA, Wang Y, Lemoine NR (2007). Expression of the IAP protein family is dysregulated in pancreatic cancer cells and is important for resistance to chemotherapy. Int J Cancer.

[17] Ruckert F, Dawelbait G, Winter C, Hartmann A, Denz A, Ammerpohl O, Schroeder M, Schackert HK, Sipos B, Kloppel G, Kalthoff H, Saeger HD, Pilarsky C (2010). Examination of apoptosis signaling in pancreatic cancer by computational signal transduction analysis. PLoS One.

[18] Tecleab A, Sebti SM (2013). Depletion of K-Ras promotes proteasome degradation of survivin. Cell Cycle.

[19] Tan N, Wong M, Nannini MA, Hong R, Lee LB, Price S, Williams K, Savy PP, Yue P, Sampath D, Settleman J, Fairbrother WJ, Belmont LD (2013). Bcl-2/Bcl-xL inhibition increases the efficacy of MEK inhibition alone and in combination with PI3 kinase inhibition in lung and pancreatic tumor models. Mol Cancer Ther.

[20] Boucher MJ, Morisset J, Vachon PH, Reed JC, Laine J, Rivard N (2000). MEK/ERK signaling pathway regulates the expression of Bcl-2, Bcl-X(L), and Mcl-1 and promotes survival of human pancreatic cancer cells. J Cell Biochem.

[21] Stevenson M (2004). Therapeutic potential of RNA interference. N Engl J Med.

[22] Shankar P, Manjunath N, Lieberman J (2005). The prospect of silencing disease using RNA interference. JAMA.

[23] Yang J, Ouyang J, Ouyang L, Ouyang L, Chen Y (2013). Inhibition of cell proliferation and increase of chemosensitivity by simultaneous knockdown of XIAP and survivin in pancreatic carcinoma cells. Oncology research.

[24] Takahashi H, Chen MC, Pham H, Matsuo Y, Ishiguro H, Reber HA, Takeyama H, Hines OJ, Eibl G (2013). Simultaneous knock-down of Bcl-xL and Mcl-1 induces apoptosis through Bax activation in pancreatic cancer cells. Biochim Biophys Acta.

[25] Kunze D, Erdmann K, Froehner M, Wirth MP, Fuessel S (2013). Enhanced Inhibition of Bladder Cancer Cell Growth by Simultaneous Knockdown of Antiapoptotic Bcl-xL and Survivin in Combination with Chemotherapy. Int J Mol Sci.

[26] Rückert F, Samm N, Lehner AK, Saeger HD, Grützmann R, Pilarsky C (2010). Simultaneous gene silencing of Bcl-2, XIAP and Survivin re-sensitizes pancreatic cancer cells towards apoptosis. BMC Cancer.

[27] Kwak EL, Bang YJ, Camidge DR, Shaw AT, Solomon B, Maki RG, Ou SH, Dezube BJ, Janne PA, Costa DB, Varella-Garcia M, Kim WH, Lynch TJ (2010). Anaplastic lymphoma kinase inhibition in non-small-cell lung cancer. N Engl J Med.

[28] Lynch TJ, Bell DW, Sordella R, Gurubhagavatula S, Okimoto RA, Brannigan BW, Harris PL, Haserlat SM, Supko JG, Haluska FG, Louis DN, Christiani DC, Settleman J (2004). Activating mutations in the epidermal growth factor receptor underlying responsiveness of non-small-cell lung cancer to gefitinib. N Engl J Med.

[29] Hanahan D, Weinberg RA (2011). Hallmarks of Cancer: The Next Generation. Cell.

[30] Lowe SW, Cepero E, Evan G (2004). Intrinsic tumour suppression. Nature.

[31] Yin H, Kanasty RL, Eltoukhy AA, Vegas AJ, Dorkin JR, Anderson DG (2014). Non-viral vectors for gene-based therapy. Nature reviews Genetics.

[32] Liu X, Wang W, Samarsky D, Liu L, Xu Q, Zhang W, Zhu G, Wu P, Zuo X, Deng H, Zhang J, Wu Z, Chen X (2014). Tumor-targeted *in vivo* gene silencing via systemic delivery of cRGD-conjugated siRNA. Nucleic Acids Res.

[33] Yan R, Hallam A, Stockley PG, Boyes J (2014). Oncogene dependency and the potential of targeted RNAi-based anti-cancer therapy. Biochem J.

[34] Zorde Khvalevsky E, Gabai R, Rachmut IH, Horwitz E, Brunschwig Z, Orbach A, Shemi A, Golan T, Domb AJ, Yavin E, Giladi H, Rivkin L, Simerzin A (2013). Mutant KRAS is a druggable target for pancreatic cancer. Proc Natl Acad Sci USA.

[35] Rückert F, Aust D, Böhme I, Werner K, Brandt A, Diamandis EP, Krautz C, Hering S, Saeger H-D, Grützmann R, Pilarsky C (2012). Five primary human pancreatic adenocarcinoma cell lines established by the outgrowth method. J Surg Res.

