# Supplementary material for: Simultaneous gene silencing of KRAS and anti-apoptotic genes as a multitarget therapy
Source: Oncotarget. 2015 Dec 26;7(4):3984–92. doi: 10.18632/oncotarget.6766 (PMC4826184; doi:10.18632/oncotarget.6766)
Supplement: Supplementary file 1 [file oncotarget-07-3984-s001.pdf]

Simultaneous gene silencing of *KRAS* and anti-apoptotic genes as a multitarget therapy

Supplementary Materials

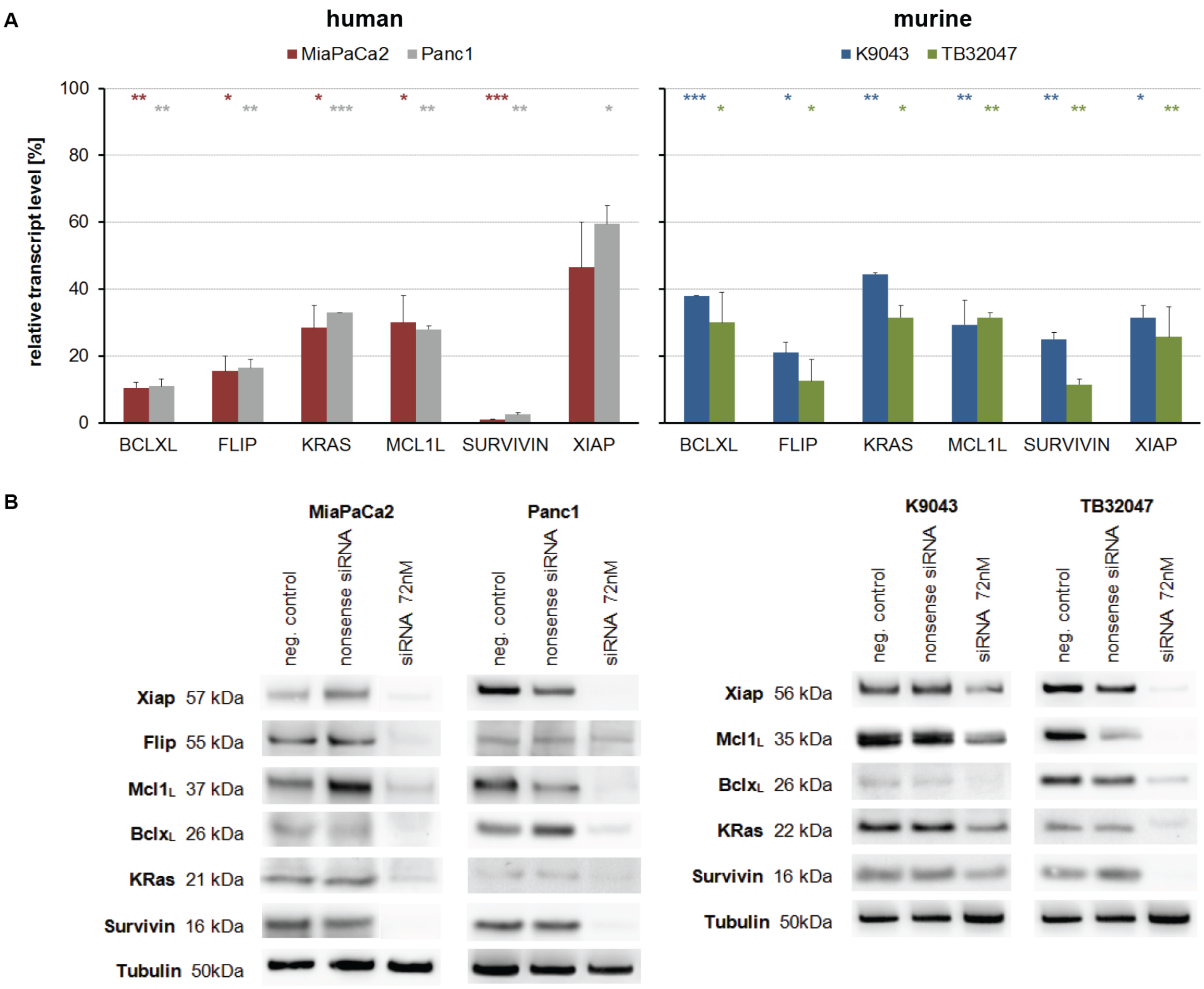

Supplementary Figure S1: Target inhibition by high-concentration siRNA treatment. Human and murine PDAC cell lines were transfected with 72 nM siRNAs for 72 h. (**A**) Transcriptional level knockdowns were confirmed by qRT-PCR. (**B**) The effect on protein quantity was confirmed by Western blotting.

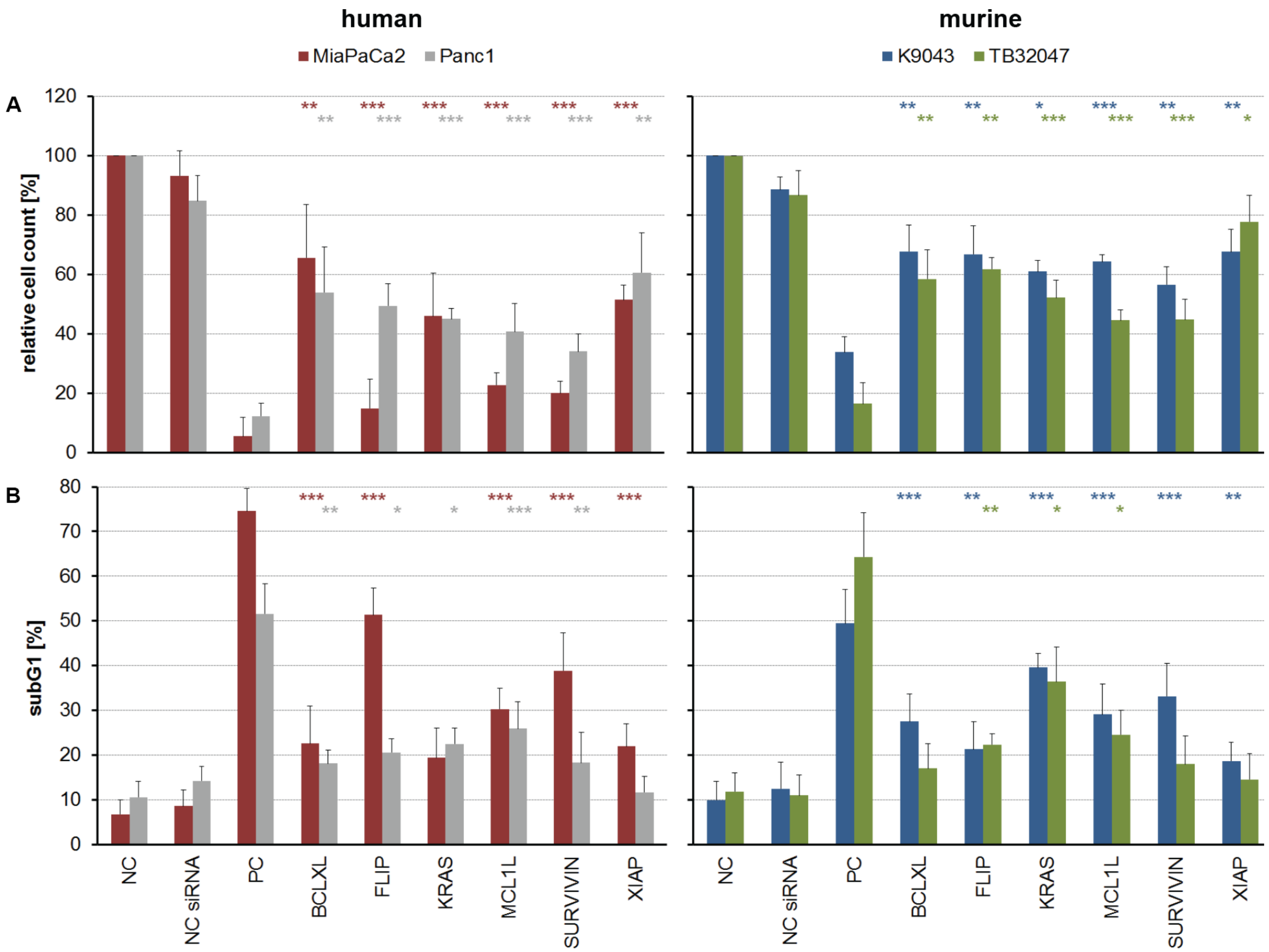

Supplementary Figure S2: Cellular effects caused by high-concentration siRNA treatment. (**A**) After gene silencing with 72 nM siRNA adherent cells were counted in relation to their negative control (NC; $n \geq 4$). (**B**) For evaluation of apoptosis induction subG1 fractions were determined by cell cycle analysis ($n \geq 4$; *$p \leq 0{,}05$, **$p \leq 0{,}01$, ***$p \leq 0{,}001$). An unpaired Student's *t*-test was used to compare the differences between the NC and the cells treated with target-directed siRNAs.

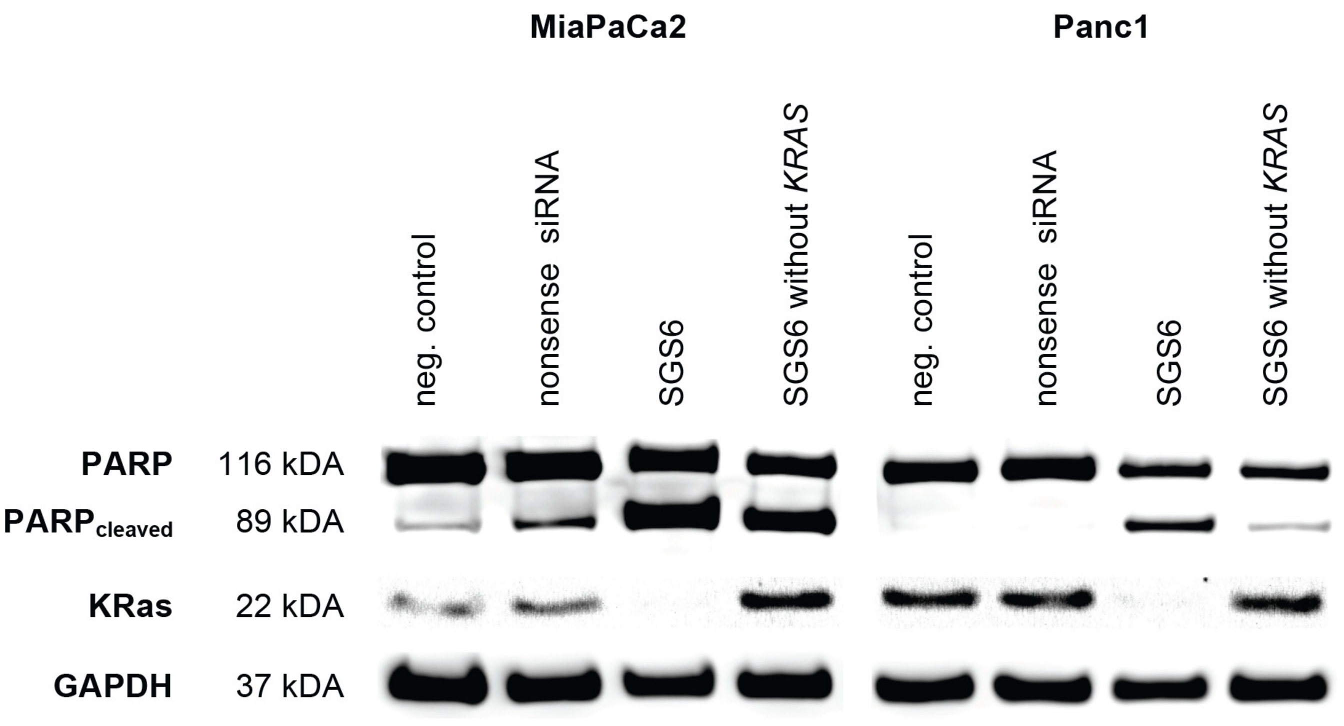

Supplementary Figure S3: Cleaved PARP induction after siRNA treatment in human pancreatic cancer cells. Cleaved PARP is induced after 72 h treatment of cells with siRNA combinations against either six (SGS6) or five (SGS6 without *KRAS*) target genes.

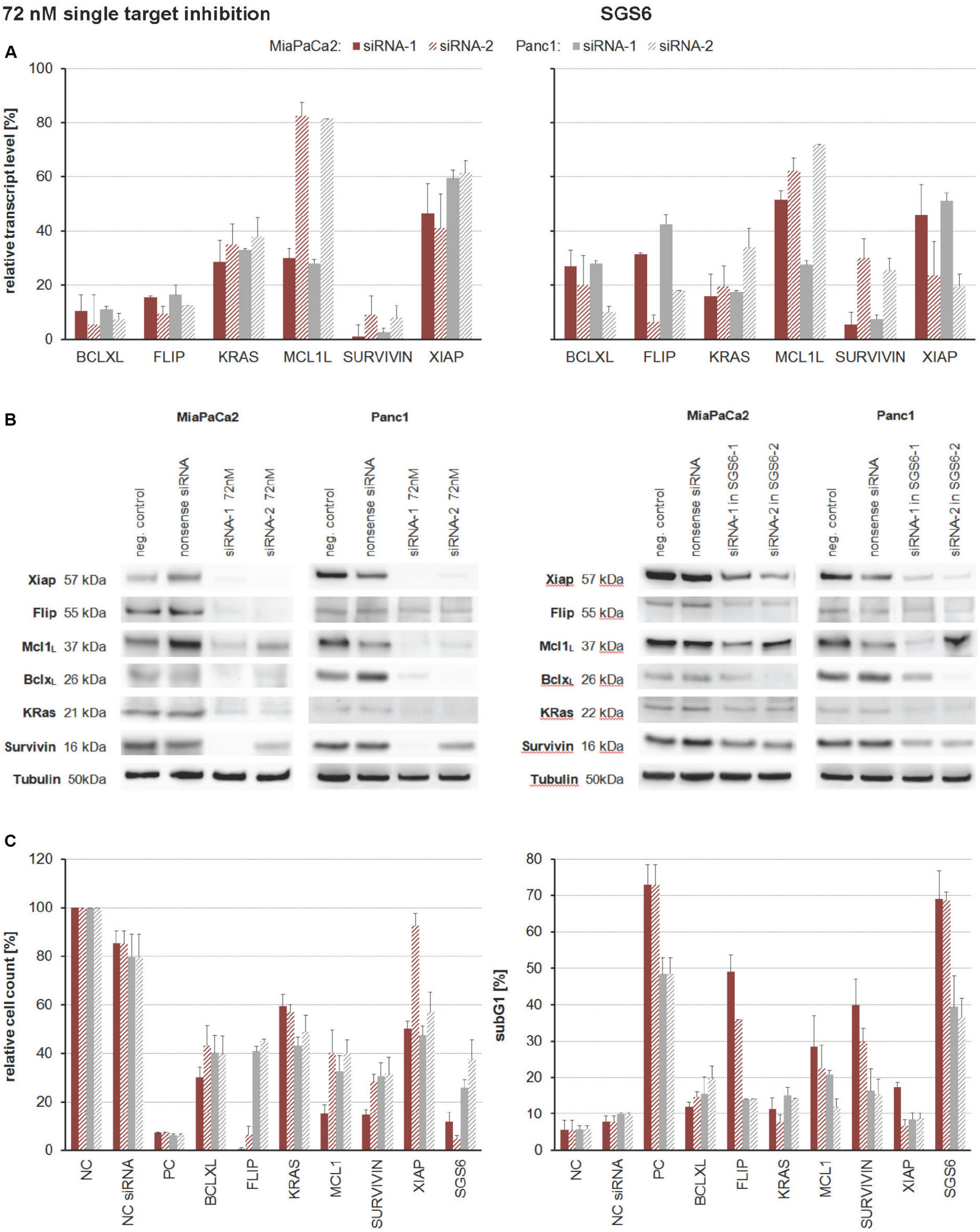

Supplementary Figure S4: Validation of gene silencing effects with second human siRNAs. Additional to the first used, human siRNAs second ones were established per gene. (**A**) Knockdowns were confirmed by qRT-PCR relative to their negative control (NC) and (**B**) by Western blots. The cellular effects were analyzed 72 h after transfection with 72 nM siRNA for single target inhibition or 6 × 12 nM for SGS6 respectively. (**C**) Adherent cells were counted and (**D**) subG1 fractions were determined by cell cycle analysis.

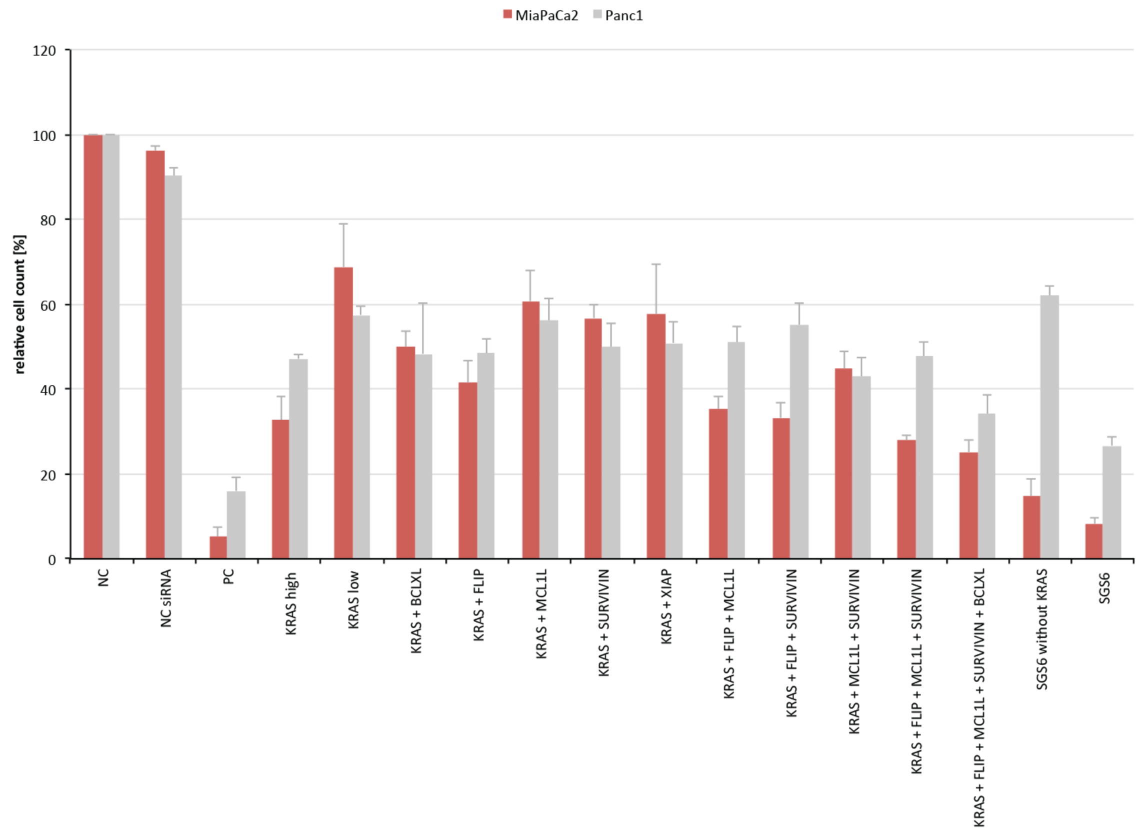

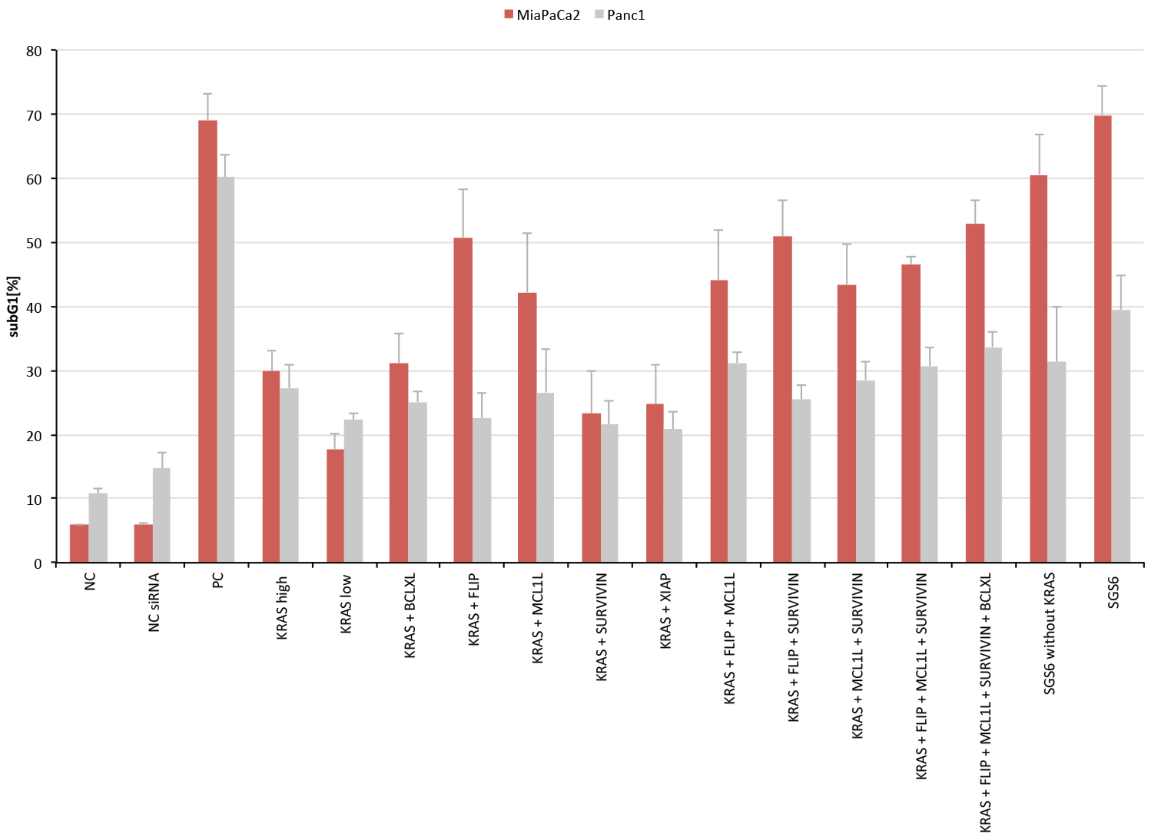

Supplementary Figure S5: Cellular effects caused by different siRNA combinations. (**A**) After gene silencing with 12 nM of each siRNA adherent cells were counted in relation to their negative control (NC; $n \geq 4$). (**B**) For evaluation of apoptosis induction subG1 fractions were determined by cell cycle analysis ($n \geq 4$).

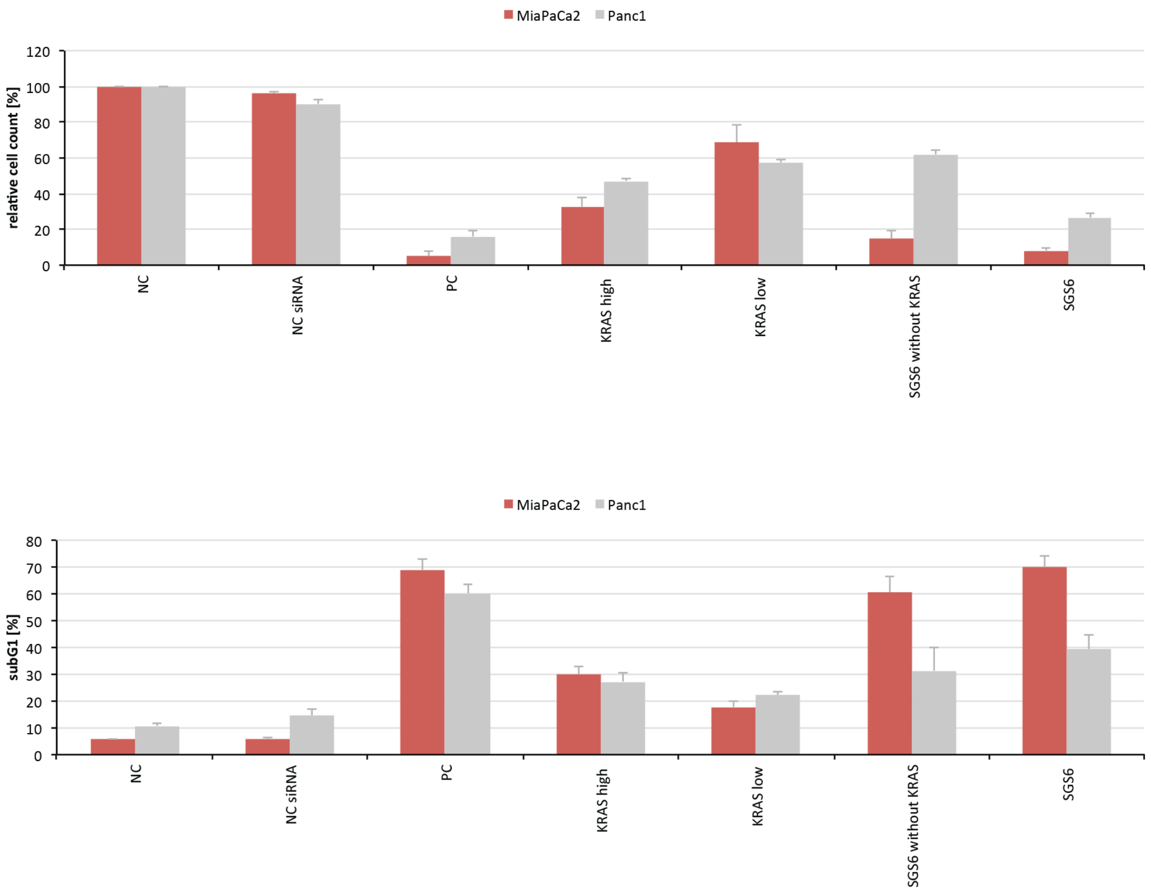

Supplementary Figure S6: Cellular effects caused by different concentrations of *KRAS* siRNA alone or in combination with five other siRNAs. (**A**) After gene silencing with 12 nM of each siRNA adherent cells were counted in relation to their negative control (NC; $n \geq 4$). (**B**) For evaluation of apoptosis induction subG1 fractions were determined by cell cycle analysis ($n \geq 4$).

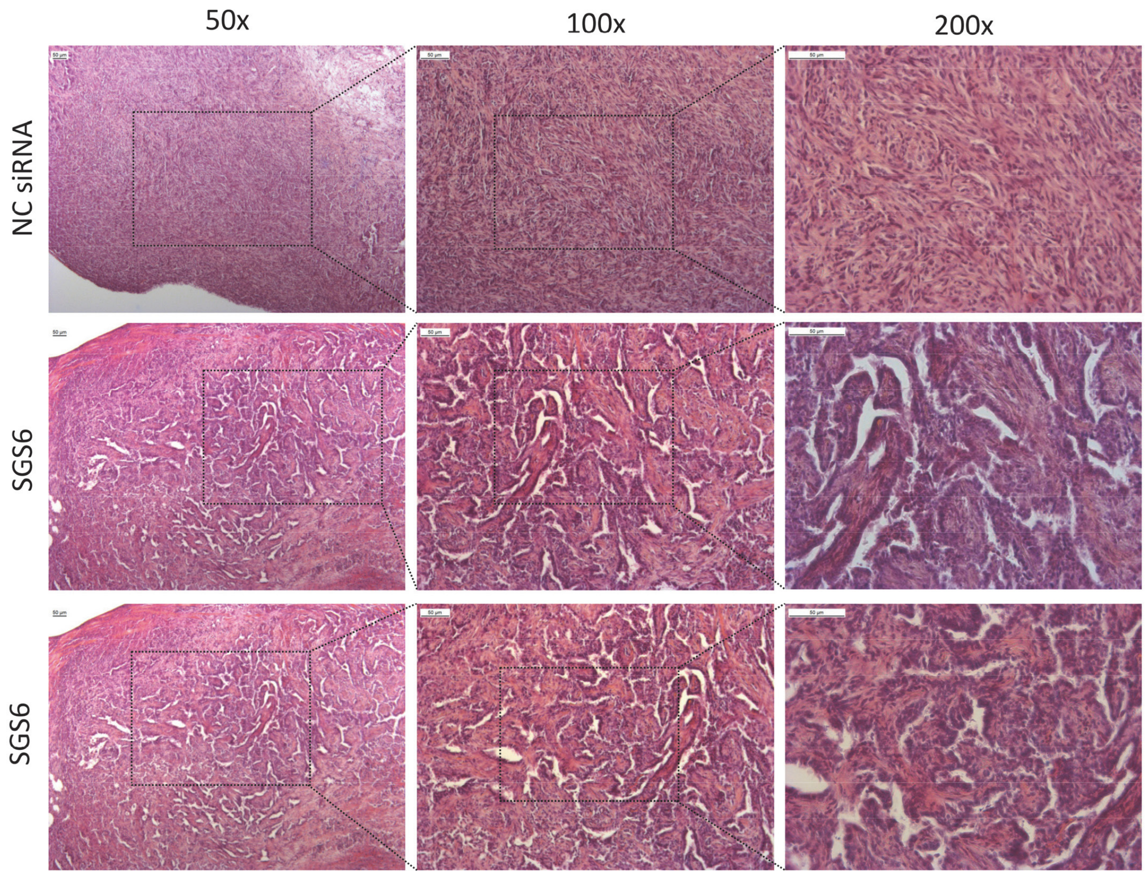

Supplementary Figure S7: Histopathological structure of subcutaneous tumors after treatment with SGS6. Upper panel: Tumor treated with allstars negative control. Middle and lower panel: tumor treated with SGS6, displaying a more differentiated glandular structure compared to the upper panel.

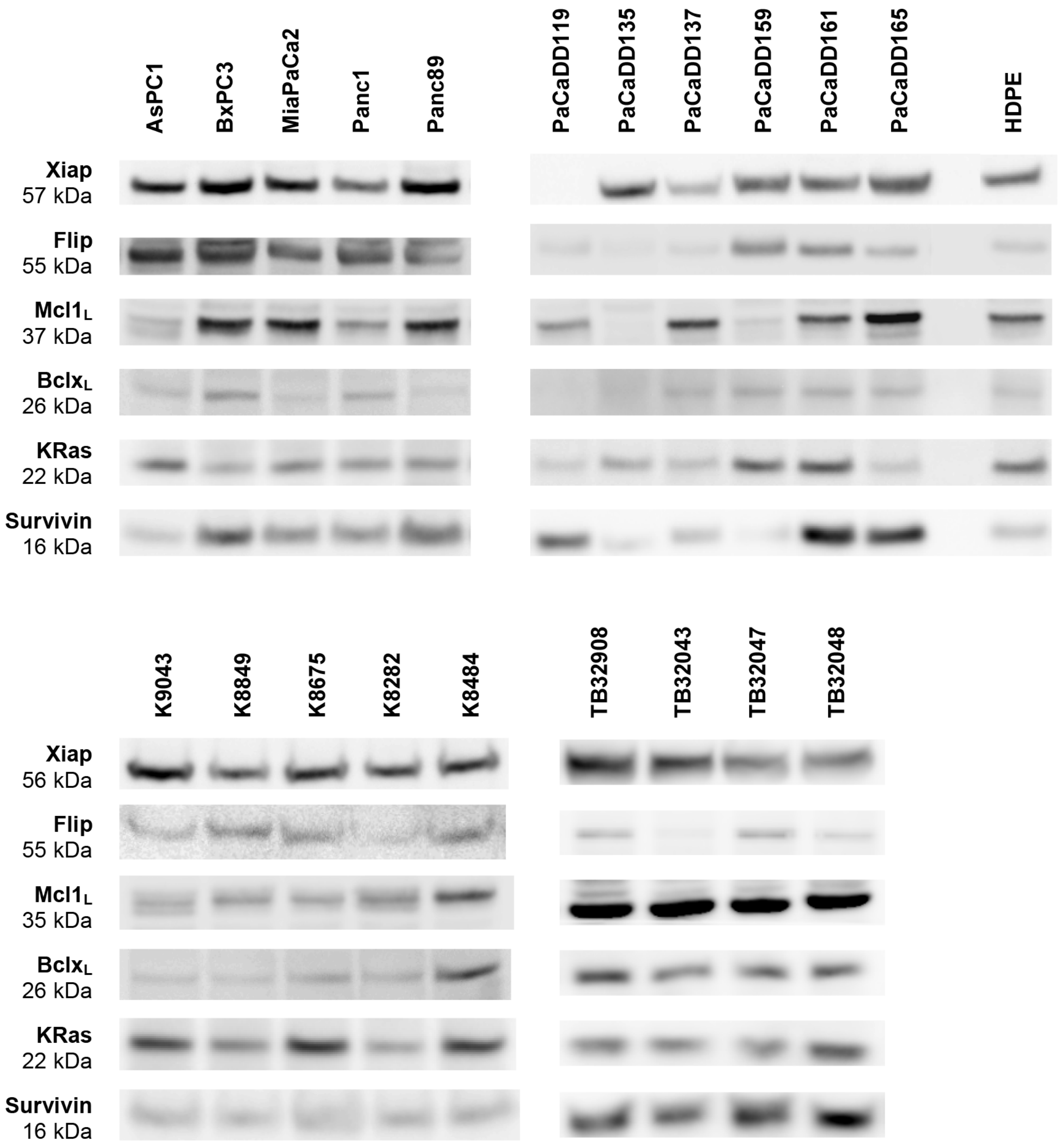

Supplementary Figure S8: Exemplary Western blots for the generation of Figure 1. Lysates of standard and primary human pancreatic cancer cell lines and from the *KPC* mouse model were subjected to Western blot analysis. Based on these analyses the expression levels of $Bclx_L$, Flip, KRas, $Mcl1_L$, Survivin and Xiap compared to the human non-tumorous, epithelial pancreatic duct HDPE-E6E7 cell line were summarized as heatmap in Figure 1.

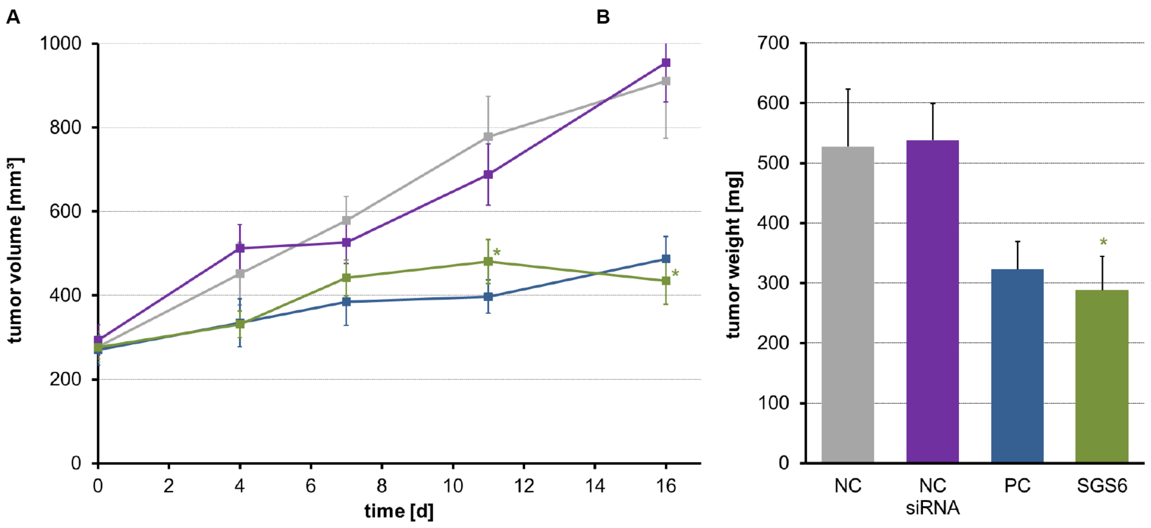

Supplementary Figure S9: Treatment of MiaPaCa2-xenografts with the SGS6 therapy. Subcutaneous, bilateral MiaPaCa2 tumors of NMRI$^{nu/nu}$ mice were treated every second day with 10 µg of *in vivo* jet-PEI complexed SGS6 siRNA for 16 d (4–6 tumors per group). (**A**) Tumor volumes were measured during therapy and (**B**) their weight was analyzed after preparation at day 18. Means ± standard errors are shown and an unpaired Student's *t*-test was used to compare the differences between the negative control (NC) and the SGS6 treated tumors (*$p \leq 0{,}05$).

Supplementary Table S1: *KRAS* mutation states, origin and grading of used cell lines

		Cell lines	Reference	*KRAS* mutation	Origin	Grading
Human	Common	HDPE-E6E7	M. Tsao	WT	pancreatic duct epithelium	–
		AsPC1	ATCC CRL-1682	G12D	ascites	G2
		BxPC3	ATCC CRL-1687	WT	primary tumor	G2
		Hs766T	ATCC HTB-134	Q61H	lymph node metastasis	G1
		MiaPaCa2	ATCC CRL-1420	G12C	primary tumor	G3
		Panc1	ATCC CRL-1469	G12D	primary tumor	G3
		Panc89/T3M4	H. Kalthoff	Q61H	lymph node metastasis	G2
	Primary	PaCaDD43	F. Rückert [1]	G12D	primary tumor	G2
		PaCaDD60		G12D	pleural effusion	G2
		PaCaDD119		G12A	primary tumor	G3
		PaCaDD135		G12V	lymph node metastasis	G2/3
		PaCaDD137		WT	primary tumor	G2
		PaCaDD159		G12V	primary tumor	G2
		PaCaDD161		G12V	liver metastasis	G3
		PaCaDD165		WT	ascites	G3
Murine	KPC	K8282	D. Tuveson [2]	G12D	*KPC* mouse model	
		K8484		G12D		
		K8675		G12D		
		K8849		G12D		
		K9043		G12D		
	KPC B6	TB32043	D. Tuveson [2]	G12D	*KPC* mouse model	
		TB32047		G12D		
		TB32048		G12D		
		TB32908		G12D		

Supplementary Table S2: Media used for the cell cultures

Cell lines	Media
AsPC1	RPMI (incl. 2 mM L-glutamine) + 10% FBS + 4,5 g/l glucose + 1 mM natrium pyruvate + 10 mM HEPES
BxPC3	RPMI (incl. 2 mM L-glutamine) + 10% FBS + 4,5 g/l glucose + 1 mM natrium pyruvate + 10 mM HEPES
HDPE-E6E7	K-SFM
Hs766T	DMEM + 10% FBS
MiaPaCa2	DMEM + 10% FBS + 2,5 % horse serum
Panc1	RPMI (incl. 2 mM L-glutamine) + 10% FBS
Panc89/T3M4	RPMI (incl. 2 mM L-glutamine) + 10% FBS
murine cell lines	DMEM + 10% FBS
primary cell lines	DMEM + 20% FBS + 50 % K-SFM

Supplementary Table S3: Designations and target sequences of the siRNAs

Target protein	Target gene	siRNA name	siRNA target sequence	Reference
$Bclx_L$	*BCL2L1*	Hs_BCL2L1.1<br>Hs/Mm_BCL2L1.2<br>Mm_BCL2L1	GCAGCUUGGAUGGCCACUU<br>AGACAAGGAGAUGCAGGUAUU<br>GCAAGUUGGAUGGCCACCU	 <br>homologous to Hs BCL2L1.1
Flip	*CFLAR*	Hs_CFLAR.1<br>Hs_CFLAR.2<br>Mm_CFLAR	CAGGAACCCUCACCUUGUU<br>AGGCAAGAUAAGCAAGGAGAA<br>GCCAAGGAGCAAGAUCAAAUA	[3]<br><br>[4]
KRas	*KRAS*	Hs_KRAS 1<br>Hs/Mm_KRAS.2	AAGGAGAAUUUAAUAAAGAUA<br>GGCUAUAUUUACAUGCUACUA	Qiagen, Hilden, Germany<br>[5]
$Mcl1_L$	*MCL1*	Hs_MCL1.1<br>Hs_MCL1.2<br>Mm_Mcl1	AAGUAUCACAGACGUUCUC<br>GAAAGCUGCAUCGAACCAU<br>GAAAGCUUCAUCGAACCAUUU	[6]<br>[7]<br>[8]
Survivin	*BIRC5*	Hs_BIRC5.1<br>Hs_BIRC5.2<br>Mm_BIRC5	GAAUUUGAGGAAACUGCGA<br>CACCACUUCCAGGGUUUAU<br>GAGUUUGAAGAGACUGCAA	[9, 10]<br>[11]<br>homologous to Hs Birc5.1
Xiap	*BIRC4*	Hs_BIRC4.1<br>Hs_BIRC4.2<br>Mm_BIRC4	GUGGUAGUCCUGUUUCAGC<br>CGAGCAGGGUUUCUUUAUA<br>GUAGUAGUCCUGUUUCAGC	[10, 12]<br>[13]<br>homologous to Hs Birc4.2
Eg5	*KIF11*	Hs/Mm_KIF11	AACUGAAGACCUGAAGACAAU	[10]

As negative control we used Allstars siRNA (Qiagen, Hilden, Germany), as positive control siRNA targeting *KIF11/Eg5*, an essential cytoskeletal component.

Supplementary Table S4: Sequences of primers for qRT-PCR

Target gene	Human		Murine	
	Primer name	Sequence (5′→3′)	Primer name	Sequence (5′→3′)
ACTB	Hs_ACTB_185_f2<br>Hs_ACTB_185_r2	AAATCTGGCACCACACCTTC<br>AGAGGCGTACAGGGATAGCA	Mm_ActB_154_f3<br>Mm_ActB_154_r3	GGCTGTATTCCCCTCCATCG<br>CCAGTTGGTAACAATGCCA<br>TGT
BCL2L1	Hs_BCL2L1_166_f1<br>Hs_BCL2L1_166_r1	ATGAACTCTTCCGGGATGG<br>TGGATCCAAGGCTCTAGGTG	Mm_BCL2L1_187_f<br>Mm_BCL2L1_187_r	GTTGGATGGCCACCTATCTG<br>AAGAGTGAGCCCAGCAGAAC
BIRC4	Hs_BIRC4_135_f1<br>Hs_BIRC4_135_r1	CACTTGAGGTTCTGGTTGCAG<br>TGCAAAGCTTCTCCTCTTGC	Mm_BIRC4_157_f<br>Mm_BIRC4_157_r	TTGGAACATGGACATCCTCA<br>TACCACTTCGCATGCTGTTC
BIRC5	Hs_BIRC5_141_f1<br>Hs_BIRC5_141_r1	GTTGCGCTTTCCTTTCTGTC<br>TCTCCGCAGTTTCCTCAAAT	Mm_BIRC5_106_f<br>Mm_BIRC5_106_r	ATCGCCACCTTCAAGAACTG<br>AATCAGGCTCGTTCTCGGTA
CFLAR	Hs_CFLAR_170_f2<br>Hs_CFLAR_170_r2	AGAGGTAAGCTGTCTGTCGG<br>TCCTCACCAATCTCTGCCAT	Mm_CFLAR_127_f<br>Mm_CFLAR_127_r	AACCCTCACCTGGTTTCTGA<br>CCTTGGCTATCTTGCCTCTG
GAPDH			Mm_GAPDH_171_f<br>Mm_GAPDH_171_r	AGCTTGTCATCAACGGGAAG<br>CGGAGATGATGACCCTTTTG
KRAS	Hs_KRAS_193_f1<br>Hs_KRAS_193_r1	GTACATGAGGACTGGGGAGG<br>TGCTAAGTCCTGAGCCTGTT	Mm_KRAS_184_f<br>Mm_KRAS_184_r	AGAGCGCCTTGACGATACAG<br>CCCTCCCCAGTTCTCATGTA
MCL1	Hs_MCL1L_175_f2<br>Hs_MCL1L_175_r2	AAGGCGCTGGAGACCTTAC<br>TCACAATCCTGCCCCAGTTT	Mm_MCL1_147_f2<br>Mm_MCL1_147_r2	CAAAGATGGCGTAACAAAC<br>TGG<br>CGTTTCGTCCTTACAAGAACA

Supplementary Table S5: Primary antibodies used for Western blots (WB)

Target protein	Manufacturer, Cat. number (Clone)	Source	Species	Dilution for WB
$Bclx_L$	QED Bioscience, 11017	mouse	Hs	1:100
	Santa Cruz, sc-634	rabbit	Hs, Mm	1:200
	CellSignaling, #2764	rabbit	Hs, Mm	1:1000
Xiap	Becton Dickinson, 610716	mouse	Hs, Mm	1:500
Survivin	CellSignaling, #2808	rabbit	Hs, Mm	1:1000
Flip	Alexis Biochemicals, ALX-804-127 (Dave2)	rat	Hs	1:1000
	Adipogene, AG-20B-0056 (NF6)	mouse	Hs	1:1000
GAPDH	CellSignaling, #2118	rabbit	Hs, Mm	1:1000
KRas	Santa Cruz, sc-30	mouse	Hs, Mm	1:100
$Mcl1_L$	Santa Cruz, sc-819	rabbit	Hs	1:100
	CellSignaling, #5453	rabbit	Mm	1:1000
PARP	CellSignaling, #9542	rabbit	Hs, Mm	1:1000
Tubulin	Sigma-Aldrich, T9026 (DM1A)	mouse	Hs, Mm	1:5000

REFERENCES

1. Rückert F, Aust D, Böhme I, Werner K, Brandt A, Diamandis EP, Krautz C, Hering S, Saeger HD,Grützmann R, Pilarsky C. Five Primary Human Pancreatic Adenocarcinoma Cell Lines Established by the Outgrowth Method. J Surg Res. 2011; 172:29–39.
2. Olive KP, Jacobetz MA, Davidson CJ, Gopinathan A, McIntyre D, Honess D, Madhu B, Goldgraben MA, Caldwell ME, Allard D, Frese KK, Denicola G, Feig C, et al. Inhibition of Hedgehog signaling enhances delivery of chemotherapy in a mouse model of pancreatic cancer. Science. 2009; 324:1457–1461.
3. Haag C, Stadel D, Zhou S, Bachem MG, Moller P, Debatin KM, Fulda S. Identification of c-FLIP(L) and c-FLIP(S) as critical regulators of death receptor-induced apoptosis in pancreatic cancer cells. Gut. 2011; 60:225–237.
4. Cleary MA, Kilian K, Wang Y, Bradshaw J, Cavet G, Ge W, Kulkarni A, Paddison PJ, Chang K, Sheth N, Leproust E, Coffey EM, Burchard J, et al. Production of complex nucleic acid libraries using highly parallel in situ oligonucleotide synthesis. Nat Methods. 2004; 1:241–248
5. Paddison PJ, Silva JM, Conklin DS, Schlabach M, Li M, Aruleba S, Balija V, O'Shaughnessy A, Gnoj L, Scobie K, Chang K, Westbrook T, Cleary M, et al. A resource for large-scale RNA-interference-based screens in mammals. Nature. 2004; 428:427–431.
6. Schulze-Bergkamen H, Fleischer B, Schuchmann M, Weber A, Weinmann A, Krammer PH, Galle PR. Suppression of Mcl-1 via RNA interference sensitizes human hepatocellular carcinoma cells towards apoptosis induction. BMC Cancer. 2006; 6: 232.
7. Machida YJ, Chen Y, Machida Y, Malhotra A, Sarkar S, Dutta A. Targeted comparative RNA interference analysis reveals differential requirement of genes essential for cell proliferation. Mol Biol Cell. 2006; 17:4837–4845.
8. Oishi K, Kamakura S, Isazawa Y, Yoshimatsu T, Kuida K, Nakafuku M, Masuyama N, Gotoh Y. Notch promotes survival of neural precursor cells via mechanisms distinct from those regulating neurogenesis. Dev Biol. 2004; 276:172–184.
9. Asanuma K, Tsuji N, Endoh T, Yagihashi A, Watanabe N. Survivin enhances Fas ligand expression via upregulation of specificity protein 1-mediated gene transcription in colon cancer cells. J Immunol. 2004; 172:3922–3929.
10. Rückert F, Samm N, Lehner AK, Saeger HD, Grützmann R, Pilarsky C. Simultaneous gene silencing of Bcl-2, XIAP and Survivin re-sensitizes pancreatic cancer cells towards apoptosis. BMC Cancer. 2010; 10:379.
11. Kenny GD, Kamaly N, Kalber TL, Brody LP, Sahuri M, Shamsaei E, Miller AD, Bell JD. Novel multifunctional nanoparticle mediates siRNA tumour delivery, visualisation and therapeutic tumour reduction in vivo. J Control Release. 2011; 149: 111–116.
12. Chawla-Sarkar M, Bae SI, Reu FJ, Jacobs BS, Lindner DJ, Borden EC. Downregulation of Bcl-2, FLIP or IAPs (XIAP and survivin) by siRNAs sensitizes resistant melanoma cells to Apo2L/TRAIL-induced apoptosis. Cell Death Differ. 2004; 11: 915–923.
13. Kunze D, Kraemer K, Erdmann K, Froehner M, Wirth MP, Fuessel S. Simultaneous siRNA-mediated knockdown of antiapoptotic BCL2, Bcl-xL, XIAP and survivin in bladder cancer cells. Int J Oncol. 2012; 41:1271–1277.
